# Redistribution of Carbon Flux toward 2,3-Butanediol Production in *Klebsiella pneumoniae* by Metabolic Engineering

**DOI:** 10.1371/journal.pone.0105322

**Published:** 2014-10-20

**Authors:** Borim Kim, Soojin Lee, Daun Jeong, Jeongmo Yang, Min-Kyu Oh, Jinwon Lee

**Affiliations:** 1 Department of Chemical and Biomolecular Engineering, Sogang University, Seoul, South Korea; 2 Department of Chemical and Biological Engineering, Korea University, Seoul, Republic of Korea; Missouri University of Science and Technology, United States of America

## Abstract

*Klebsiella pneumoniae* KCTC2242 has high potential in the production of a high-value chemical, 2,3-butanediol (2,3-BDO). However, accumulation of metabolites such as lactate during cell growth prevent large-scale production of 2,3-BDO. Consequently, we engineered *K. pneumoniae* to redistribute its carbon flux toward 2,3-BDO production. The *ldhA* gene deletion and gene overexpression (*budA* and *budB*) were conducted to block a pathway that competitively consumes reduced nicotinamide adenine dinucleotide and to redirect carbon flux toward 2,3-BDO biosynthesis, respectively. These steps allowed efficient glucose conversion to 2,3-BDO under slightly acidic conditions (pH 5.5). The engineered strain SGSB105 showed a 40% increase in 2,3-BDO production from glucose compared with that of the host strain, SGSB100. Genes closely related to 2,3-BDO biosynthesis were observed at the gene transcription level by cultivating the SGSB100, SGSB103, SGSB104, and SGSB105 strains under identical growth conditions. Transcription levels for *budA*, *budB*, and *budC* increased approximately 10% during the log phase of cell growth relative to that of SGSB100. Transcription levels of 2,3-BDO genes in SGSB105 remained high during the log and stationary phases. Thus, the carbon flux was redirected toward 2,3-BDO production. Data on batch culture and gene transcription provide insight into improving the metabolic network for 2,3-BDO biosynthesis for industrial applications.

## Introduction

Methods for engineering cell metabolism have enabled the production of high-value chemical compounds in large quantities. A single cell can be engineered to convert various carbon sources into valuable biochemical products such as lactate, butadiene, succinate, and 2,3-butanediol (2,3-BDO) [1], [2]. Among these products, 2,3-BDO is essential for the synthesis of various biosynthetic products of economic value. For example, 2,3-BDO can be converted into C_4_ industrial-platform chemicals such as methyl ethyl ketone and 1,3-butadiene [3], [4].

*Klebsiella pneumoniae* is a very promising microorganism for 2,3-BDO production. However, as shown in previous studies, the conversion ratio of glucose into 2,3-BDO varies with pH and temperature [5]–[7]. The pathway for 2,3-BDO synthesis competes with the pathway for organic acid production. As 2,3-BDO production increases at low pH, it prevents acidification of the medium [8], [9]. In addition, previous studies have confirmed that the enzyme activity of genes related to 2,3-BDO production is elevated at 37°C [10]. However, even under optimized conditions for culture growth, the 2,3-BDO conversion ratio still cannot reach the optimal theoretical yield.

The ratio of carbon conversion into the target product is a very important factor in cell metabolic engineering. To increase the 2,3-BDO production ratio, genetic engineering for the redistribution of carbon flux toward 2,3-BDO synthesis is necessary. Recently, many investigators have attempted to increase 2,3-BDO production of various microorganisms using various methods such as metabolic engineering and medium optimization. For example; optimal medium was determined using the two-level Plackett-Burman design with a newly isolated *Klebsiella pneumonia* achieving 150 g/L 2,3-BDO concentration (productivity: 4.21 g/L.h, yield: 0.43 g/g) by fed-batch fermentation when glucose is the carbon source [11], mutants deficient in ethanol formation were constructed by replace the aldA gene coding for aldehyde dehydrogenase with a tetracycline resistance cassette in *Klebsiella oxytoca* achieving 130 g/L 2,3-BDO concentration (productivity: 1.64 g/L.h, yield: 0.48 g/g) by fed-batch fermentation when glucose is the carbon source [12], the gene encoding lactate dehydrogenase was deleted in *Enterobacter aerogenes* achieving 118 g/L 2,3-BDO concentration (productivity: 3.20 g/L.h, yield: 0.42 g/g) by fed-batch fermentation when glucose is the carbon source [13], optimal medium was determined combining the Plackett-Burman design and response surface methodology with *Serratia marcescens* achieving 139 g/L 2,3-BDO concentration (productivity: 3.49 g/L.h, yield: 0.47 g/g) by fed-batch fermentation when sucrose is the carbon source [14], Co-overexpression of bdh (2,3-BDO dehydrogenase) and gapA (glyceraldehyde-3-phosphate dehydrogenase) genes were done in *Bacillus amyloliquefaciens* achieving 132 g/L 2,3-BDO concentration (productivity: 2.95 g/L.h, yield: 0.45 g/g) by fed-batch fermentation when glucose is the carbon source [15]. Also, Some strains which do not produce 2,3-BDO were engineered to possible producing 2,3-BDO [16], [17]. These studies are focused on increasing the titer and yield of 2,3-BDO in accordance with the carbon consumption of the microorganism, and they have achieved a sufficient titer and yield of 2,3-BDO. However, further research at the transcription level is required in order to understand the distribution of carbon flux leading to 2,3-BDO biosynthesis in microorganisms. Comparing the maximum productivity (2,3-BDO g/l.h) of various mutants is also important to determine the possibility of practical industrial applications.

**Figure 1:**
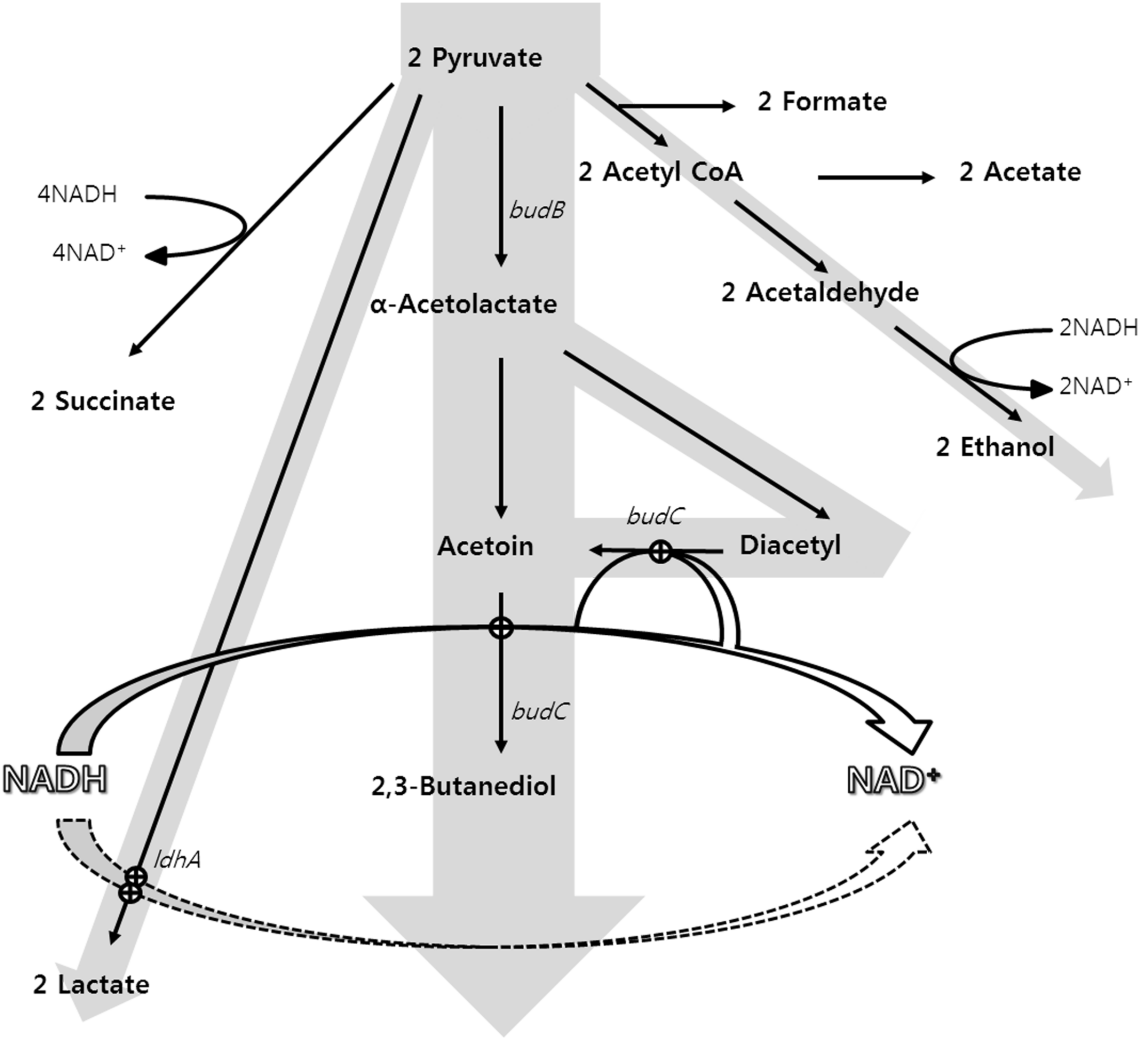
Major metabolic pathways in *K. pneumoniae* that produce byproducts, including 2,3-BDO. Gray arrows and bold arrows indicate carbon flux and enhanced metabolic pathways due to *budA* and *budB* gene overexpression, respectively.

In the present study, the following three mutants were constructed through genetic engineering of the SGSB100 strain of *K. pneumoniae* (*wabG* gene deleted strain): a strain with its lactate dehydrogenase gene (*ldhA*) deleted (SGSB103), a strain overexpressing the acetolactate decarboxylase (*budA*) and acetolactate synthase (*budB*) genes (SGSB104) and, a strain with *ldhA* deletion as well as *budA* and *budB* overexpression (SGSB105) (Fig. 1). The carbon flux distribution of the three strains was compared by culturing the microorganisms under identical fermentation conditions. To observe the genetic changes due to the carbon flux distribution, genes closely related to 2,3-BDO biosynthesis were analyzed at the gene transcription level.

## Materials and Methods

### Strains and plasmids

Strains, plasmids, and primers used in the study are listed in Table 1. The *budA* and *budB* genes were derived from *K. pneumoniae* KCTC2242 (Korean Collection for Type Culture). *Escherichia coli* DH5α [F-(80d lacZ M15) (lacZYA-rgF)U1691 hsdR17(m+)recA1 endA1 relA1 deoR; Dong In Biotech Co., Korea] was used for the manipulation of plasmids and for gene cloning. The pUC18 plasmid (Takara Shuzo Co., Ltd., Japan) containing the *lacZ* promoter was used to clone the *budA* and *budB* genes, and the pET28a plasmid (Takara Shuzo Co., Ltd., Japan) was used to clone the kanamycin resistance gene into the pUC18 vector.

**Table 1:** Bacterial strains and plasmids used in this study.

Strain or plasmid	Genotype or properties	Source
**Strains**		
*Escherichia coli* DH5α	F- endA1 glnV44 thi-1 recA1 relA1 gyrA96 deoR nupG Φ80d*lacZ*ΔM15 Δ(*lacZYA-argF*)U169, hsdR17(r_K_^−^ m_K_^+^), λ−	RBC
*Klebsiella pneumoniae* KCTC2242	Ap^r^, *trp*R	KCTC
SGSB100	*K. pneumoniae* KCTC2242 Δ*wabG*	[19]
SGSB103	*K. pneumoniae* KCTC2242 Δ*wabG*Δ*ldhA*	This study
SGSB104	*K. pneumoniae* KCTC2242 Δ*wabG*::*budA*::*budB*	This study
SGSB105	*K. pneumoniae* KCTC2242 Δ*wabG*Δ*ldhA*::*budA*::*budB*	This study
**Plasmids**		
pUC18	Ap^r^	Takara
pET-28a	Kan^r^	Takara
pUC18K	Ap^r^, pUC18 containing Kan^r^ from pET-28a	This study
pRedET	Tet^r^, containing *gam*, *exo*, and *bet* genes	Gene Bridges
**Primers** a		
PR1	5′-**GAATTC**AGGAAGTGGTATATGAATCATTCTGC-3′ (*Eco*RI)	This study
PR2	5′-**GAGCTC**TTAACTTTCTACGGAACGGATGG-3′(*Sac*I)	This study
PR3	5′-**GAGCTC**ATGGACAAACAGTATCCGGTAC-3′ (*Sac*I)	This study
PR4	5′-**GGATCC**TTACAGAATCTGACTCAGATGCAG-3′ (*Bam*HI)	This study

a The bold letters denote the restriction sites.

### Enzymes and chemicals

A DNeasy tissue kit (Qiagen) was used to isolate genomic DNA from *K. pneumoniae* KCTC2242. Restriction enzymes and T4 DNA ligase were purchased from Takara Shuzo. An Axy PrepTM kit (Axygen) was used to isolate bacterial plasmid DNA. A gel extraction kit (Takara, A550) was used for large-scale isolation of plasmid DNA from gels.

### Plasmid construction and gene deletion

Sequence information for the *budA, budB* and *ldhA* genes in *K. pneumoniae* KCTC2242 were provided by the National Center for Biotechnology Information Genbank (CP002910.1) [18]. Kyung Hee University (Seoul, Korea) provided the SGSB100 strain, which had a *wabG* gene deletion [19].

For the *ldhA* gene deletion, pRedET and 707-FLPe plasmids (Gene Bridge, Germany) were used for homologous recombination. The polymerase chain reaction (PCR) cycles were as follows: 95°C for 5 min, followed by 25 cycles at 95°C for 30 s, 66°C for 55 s, and 72°C for 60 s. FRT was introduced into the cassette in order to remove the kanamycin selection marker from the chromosome after disrupting the target gene,. This was accomplished by transforming the cells with the FLP expression plasmid. The PCR products (FRT-flanked kanamycin-resistance gene, FCF) were ligated into the pGEM-T Easy vector (Promega, USA). Subsequently, FCF was used as the template for PCR reaction with a primer pair containing 50 bp homologous sequences at their 5′ extremities that corresponded to both ends of *ldhA*. The sequences of the primer pair for strain SGSB100 were as follows (homologous sequences are indicated in italics): *ldhA*/forward, 5′-*ATGAAAATCGCGGTTTATAGTACGAAGCAGTACGATAAAAAGTACCTGCA*AATTAACCCTCACTAAAGGGCG-3′; and *ldhA*/reverse, 5′-*TTAGACGATGGCGTTCGGACAGGTTTCGCCGTTGGCGACCTGCTGCAGGT* TAATACGACTCACTATAGGGCTCGA-3′. The final PCR product contained the kanamycin resistance gene flanked by FRT and 50 bp sequences homologous to both ends of the *ldhA* gene (LFKFL). The PCR cycles were as follows: 95°C for 5 min followed by 25 cycles at 95°C for 30 s, 65°C for 60 s, and 72°C for 60 s. Purification of plasmid DNA and PCR products were performed with a Qiagen plasmid kit (Qiagen) and a PCR purification kit (Qiagen). All PCR experiments were carried out with 0.6 U of Taq polymerase (Takara, Japan).

To effect overexpression of the *budA* gene, PR1 and PR2 primers were used to clone the *budA* gene at a melting temperature (Tm) of 58.0°C. The cloned *budA* gene fragment within the pUC18K vector was inserted into the *Eco*RI and *Sac*I restriction sites. For *budB* gene overexpression, PR3 and PR4 primers were similarly used to clone the *budA* gene at a Tm of 58.0°C. The cloned *budB* gene fragment within the pUC18K vector harboring *budA* was inserted into the *Sac*I and *Bam*HI restriction sites. Plasmids were then transformed into competent cells prepared from the SGSB100 or SGSB103 strains. Electroporation (Gene Pulser; Bio-Rad) of 50 µl of cell suspensions with 1 µl of DNA in 0.2 cm gap cuvettes was performed at 2.48 kV, 25 µF, and 200 Ω.

### Culture conditions

Stock cultures of SGSB100 and mutant strains were grown in LB medium containing 10 g of tryptone (Difco, Detroit, MI, USA), 5 g of yeast extract (Difco), and 10 g of NaCl (all in quantities per liter of deionized water at pH 7.2, unless otherwise stated). Media requiring solidification contained 20 g/L agar. Selection was performed in media with added ampicillin (50 µg/ml) and kanamycin (50 µg/ml). To prepare the batch culture, the stock culture was inoculated into a 2.5 L fermenter (Biotron, South Korea) at 10% inoculation volume and 1 L operating volume. Recombinant clones were cultivated in minimal medium containing 4 g of sodium sulfate, 5.36 g of ammonium sulfate, 1.0 g of ammonium chloride, 7.3 g of dipotassium phosphate, 1.8 g of monosodium dihydrogen phosphate monohydrate, 1.2 g of ammonium citrate, 4.0 g of magnesium sulfate, 0.02 g of thiamine, and 3% of carbon source (all in quantities per liter of deionized water at pH 7.2, unless otherwise stated). Selection was performed in media with added ampicillin (50 µg/ml) and kanamycin (50 µg/ml). Dissolved oxygen was provided by injection of filtered air at a flow rate of 1.5 vvm, and the agitation speed was maintained at 200 rpm. The pH was maintained at 6.5 through automatic addition of 5 N NaOH and 5 N HCl solutions. All batch cultures were repeated three times. In the fed-batch fermentation, 1 L of glucose solution (700 g/L) was fed into the bioreactor at a rate of 3.5 ml/min at 30 min intervals. Culture growth conditions were the same as those of the batch culture with the exception of having a pH of 5.5.

### RNA extraction, reverse transcription, and real-time PCR (RT-PCR)

Samples were withdrawn periodically and then centrifuged at 4,500 rpm for 2 min at 4°C. For total DNA extraction, 1 ml of RNAiso PLUS reagent (Takara, Japan) was added to each suspension of 5×10^6^ cells. Subsequently, the resuspended cells were left at room temperature for 5 min to isolate the RNA from the nuclear protein. Subsequently, chloroform of 0.2 times the amount of RNAiso Plus used was added to the suspension. The mixture was again allowed to stand at room temperature for 5 min and then centrifuged at 4,500 rpm for 15 min at 4°C. The top layer of the supernatant was collected, isopropanol at 0.5 times the volume of the supernatant was added, and then the mixture was allowed to stand for 10 min at room temperature. The solution was centrifuged at 4,500 rpm for 10 min at 4°C to precipitate the RNA. All of the RNA was dissolved in RNase-free water.

To synthesize the complementary DNA of each RNA sample, PrimeScript reverse transcriptase (Takara, Japan) was used. First, a mixture of isolated RNA, Oligo(dT) primer, dNTP, and RNase-free water was incubated at 65°C for 5 min. Second, PrimeScript reverse transcriptase, 5× PrimeScript buffer, and RNase inhibitor were added to the above solution, and the resulting mixture was incubated at 42°C for 60 min. Finally, the mixture was heated at 70°C for 15 min and then cooled on ice.

The reactant thus obtained was used as the template for the RT-PCR experiment. PrimeScript RT Master Mix (Takara, Japan) and a Thermal Cycler Dice Real Time System Lite (Model TP700/760, Software version: V5.0x, Takara, Japan) were used for the RT-PCR experiment and for ΔΔCt analysis. Primers used in the RT-PCR experiment are listed in Table 2.

**Table 2:** Primers used in real-time PCR.

Primer	Sequence
budR-FW	GGTATTTCACAGCCTCCGTT
budR-Rev	AGAAAGACTCTCCCGCTTCC
budB-FW	TCTCACCGATAACTTTGGCA
budB-Rev	GCCAGGTAGTACCAGAAGCC
budA-FW	GACCGACGTACTCGACGAT
budA-Rev	TTGCGGTCATCGGTAATAAA
budC-FW	ACAACATCAACGTCAAAGGG
budC-Rev	GGAACAGGCGTTGATGATTT
ldhA-FW	TTGCGAAGCGGTATGTATCT
ldhA-Rev	AGGTCGACGTTGTTAAACCC
ack-FW	GGTGGTTCTGTTTCCGCTAT
ack-Rev	GTGCAGATGGAAGATGATGG
16srRNA-FW	AGAGTTTGATCMTGGCTCAG
16srRNA -Rev	GGTTACCTTGTTACGACTT

### Analytical methods

Samples were withdrawn periodically and then centrifuged at 4,500 rpm for 10 min. The amount of carbon source, 2,3-BDO, and organic acids in the supernatants were measured on a high-performance liquid chromatography (HPLC) system using an RI2414 detector (Waters Co., USA) and an Aminex HPX-87H organic acid column (300×7.8 mm; Bio-Rad). Sulfuric acid solution (0.01 M) was used as the mobile phase. The temperature was 60°C and the flow rate was 0.6 ml/min. All solutions were filtered through a 0.2 µm membrane before use. .

## Results

### Construction of *ldhA*-deficient strains and of strains overexpressing the *budA* and *budB* genes

Since 2,3-BDO biosynthesis is a downstream metabolic process originating with pyruvate, increasing the pyruvate flux toward 2,3-BDO biosynthesis was the key strategy to obtain a high yield of 2,3-BDO.

Accordingly, two approaches were established. First, the *ldhA* gene, which is the main gene related to lactate biosynthesis, was deleted to block the carbon flux toward lactate production and to minimize the consumption of reduced nicotinamide adenine dinucleotide (NADH) during lactate biosynthesis. Among the five genes related to lactate biosynthesis (*lldD*, *ldhD*, *ldhL*, *ldhA*, and *dld*), *ldhA* was identified by Macrogen Inc. (Seoul, South Korea) as a key gene for lactate production in *K. pneumoniae* KCTC2242. This gene was deleted through homologous recombination to produce the SGSB103 strain. Second, we constructed a pUC18K vector harboring the *budA* and *budB* genes and transported it into the SGSB100 strain to produce the SGSB104 strain. This approach was based on the effect of overexpression of *budA*, *budB*, and *budC* genes on 2,3-BDO biosynthesis reported in a study by Kim et al. (2012). They observed that the mutant overexpressing *budA* and *budB* genes showed the highest 2,3-BDO production yield.

A third strain SBSG105 was constructed through a combination of the two strategies. This was done in order to minimize the carbon flux and NADH consumption during lactate biosynthesis, as well as to redirect the carbon flux toward 2,3-BDO production by blockage of the lactate biosynthetic pathway.

### Redistribution of carbon flux toward 2,3-BDO production in batch fermentation

Batch fermentation of SGSB100, SGSB103, SGSB104, and SGSB105 strains was conducted under identical fermentation conditions: working volume of 1 L medium, 3% glucose, 37°C, pH 6.5, 200 rpm, and filtered air at a flow rate of 1.5 vvm. Batch fermentation was performed during a 24 h period, and samples of each strain were collected during the lag (3 h), log (6 h), and stationary (11, 18, and 24 h) phases. The time-course profiles of metabolites and carbon distribution at the stationary phase of the strains were obtained by HPLC and then compared.

**Figure 2:**
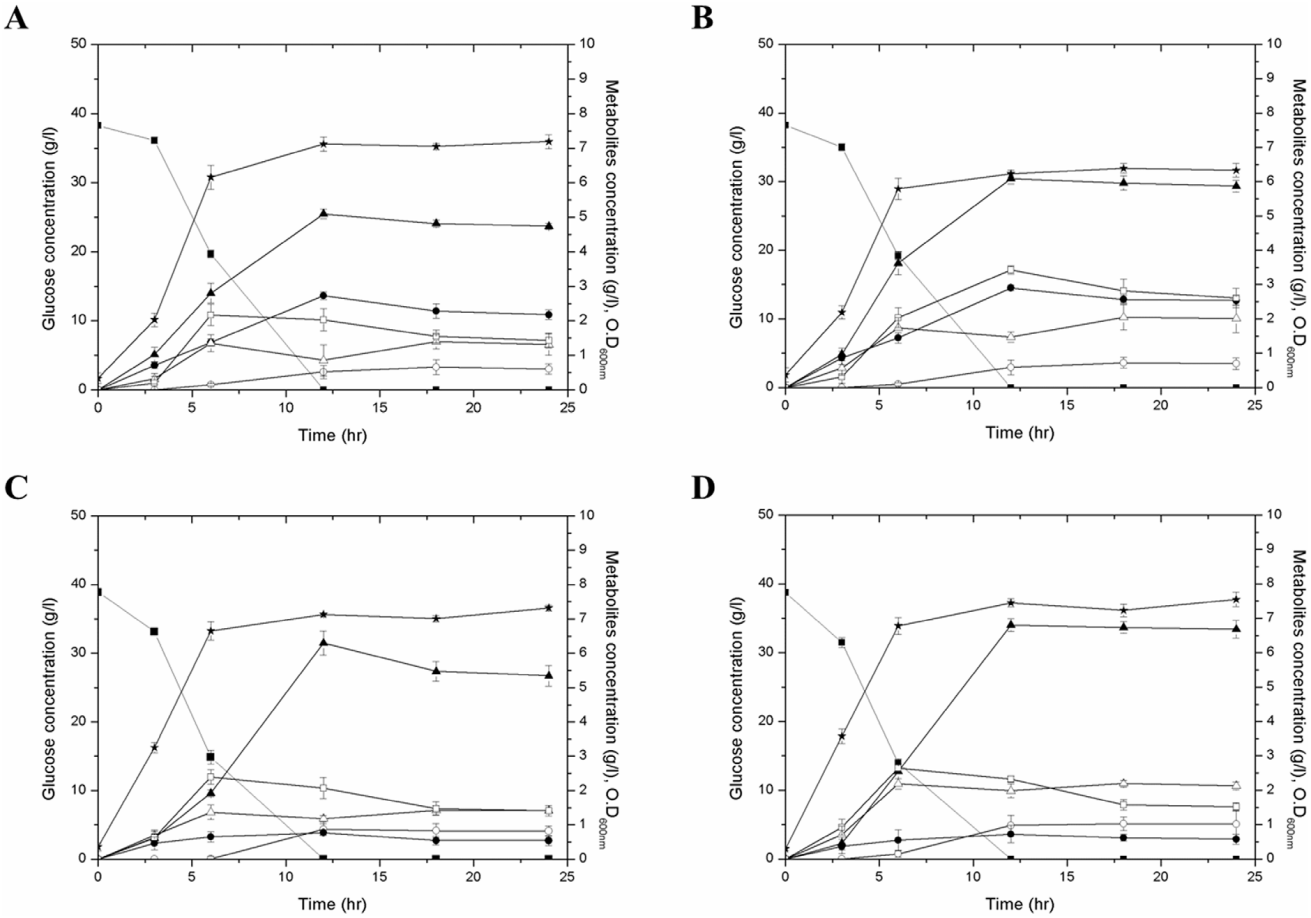
Batch fermentation profiles of metabolites in *K. pneumoniae* mutant strains: (A) SGSB100, (B) SGSB103, (C) SGSB104, and (D) SGSB105. Closed squares = glucose; closed stars = cell growth; closed triangles = 2,3-BDO; closed circles = lactate; open squares = ethanol; open triangles = acetate; open circles = succinate.

The time-course profiles of the metabolite were compared to investigate the effect of *ldhA* deletion and *budA* and *budB* overexpression in common culture conditions in batch fermentation (Fig. 2). Strains with *ldhA* deletion (SGSB103 and SGSB105) consumed glucose faster than did the other strains during the first 6 h because of rapid cell growth. However, all strains completely consumed glucose in the medium within 12 h, at which point, rates of cell growth among strains were similar. 2,3-BDO was the main product of SGSB100 metabolism, and lactate was the second major product. Similar to 2,3-BDO production, lactate production during the lag and log phases of cell growth steadily increased. However, lactate production during the stationary phase reached equilibrium whereas 2,3-BDO production rates declined.

Overall, lactate production by strains SGSB103 and SGSB105 were low (<1 g/L), and 2,3-BDO production was approximately 20–40% higher than that of SGSB100. Because of the *ldhA* gene deletion in SGSB105, the carbon flux toward lactate production was reduced to 1.6% with respect to the carbon conversion ratio. In contrast, 6.1% of carbon flux in SGSB100 is directed towards lactate production (Table 3). The maximum amounts of 2,3-BDO produced by SGSB100, SGSB103, SGSB104, and SGSB105 were 5.0, 6.1, 6.3, and 6.8 g/L, respectively. The maximum amount of 2,3-BDO produced by SGSB105 increased by 36% relative to that of SGSB100 (Fig. 2). Also, the carbon flux toward 2,3-BDO was increased to 30.2% in SGSB105 (Table 3). Byproducts produced during batch fermentation of SGSB100, SGSB103, SGSB104, and SGSB105 included succinate (0.51, 0.87, 0.59, and 0.97 g/L, respectively), acetate (0.86, 1.18, 1.47, and 1.98 g/L, respectively), and ethanol (2.03, 2.07, 3.43, and 2.33 g/L, respectively. Succinate production by all strains was low (<1g/L), but *ldhA* gene deletion strains tended to produce more succinic acid. Meanwhile, acetate production by SGSB105 was higher than other strains. Because the acetate is known to induce the production pathway of 2,3-BDO, it seems that the acetate production proportional to the amount of 2,3-BDO. Amount of ethanol production in SGSB105 was similar to host strain SGSB100.

**Table 3:** The distribution of glucose by *K, pneumoniae* SGSB100, SGSB103, SGSB104 and SGSB105 in batch fermentation at 12hr (carbon conversion ratio (%)).

Strains	Percentage (%)						
	2,3-BDO	Lactate	Acetate	Succinate	Formate	Ethanol	Cell mass
SGSB100	22.6	6.1	2.9	1.8	2.8	8.8	11.7
SGSB103	28.0	1.7	3.9	3.0	2.2	9.0	11.7
SGSB104	27.0	6.4	4.9	2.0	5.0	14.9	10.4
SGSB105	30.2	1.6	6.6	3.3	2.0	10.1	12.2

### RT-PCR assay of genes for *ldhA* and 2,3-BDO biosynthesis in mutant strains

Changes at the gene transcription level that might have resulted from the carbon flux redistribution were analyzed by RT-PCR assay. The four strains were cultivated under identical growth conditions, and mRNA samples at the lag (3 h) and stationary (12 h) phases of cell growth of each strain were obtained.

**Figure 3:**
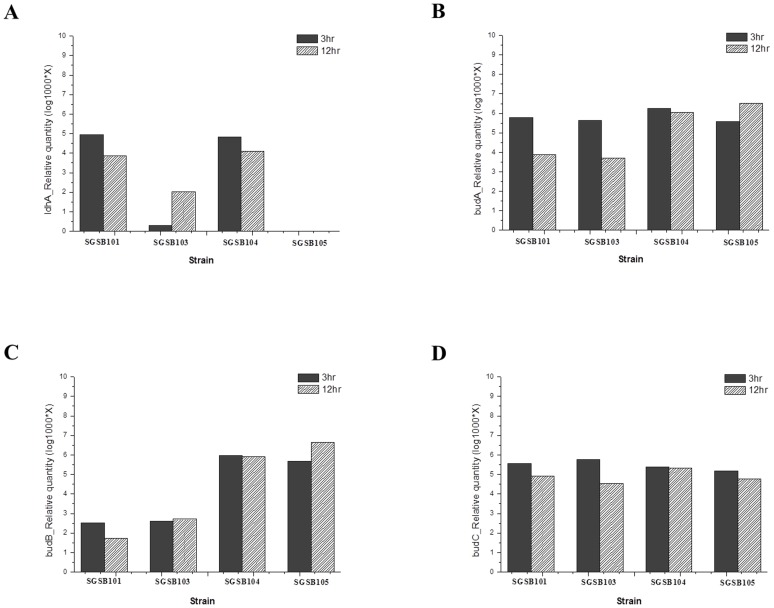
Real-time PCR results for (A) *ldhA*, (B) *budA*, (C) *budB*, and (D) *budC* genes of *K. pneumoniae* strains SGSB100, SGSB103, SGSB104, and SGSB105 at 3 h (black filling) and at 12 h (herringbone filling) of batch fermentation. The abscissa and ordinate represent strains and expression levels of each gene, respectively. X = relative quantity (second derivative maximum) based on the value for the 16srRNA gene.

Eight genes related to biosynthesis of metabolites, including 2,3-BDO (*ldhA*, *budA*, *budB*, *budC*, *budR*, *ack*, *pflB*, and *aldA*), were selected and analyzed. Among these genes, four (*ldhA*, *budA*, *budB*, and *budC*) exhibited significant changes in transcription level due to carbon flux redistribution in the mutant strains, relative to those of the SGSB100 strain. The RT-PCR results were analyzed through the ΔΔCt method using the 16srRNA gene as the reference gene. Results are presented as relative quantities (i.e., second derivative maxima, Fig. 3).

First, relative quantities (second derivative maxima) of the *ldhA* gene expression levels of the SGSB100, SGSB103, SGSB104, and SGSB105 strains at the lag and stationary phases of cell growth (9.051E+01/7.260E+00, 1.97E−03/1.07E−01, 6.86E+01/1.29E+01, and undetected, respectively) were obtained. As shown Fig. 3A, the *ldhA* gene expression level in the SGSB100 and SGSB104 strains was reduced at the stationary phase. However, the SGSB103 strain exhibited a significantly low level of *ldhA* gene expression compared with that of the SGSB100 and SGSB104 strains at both lag and stationary phases. The *ldhA* gene of the SGSB105 strain showed reduced expression level of the gene, which reflects the absence of amplification of the *ldhA* gene (Fig. 3A).

Second, the relative quantity data of the *budA* and *budB* genes in SGSB104 and SGSB105 increased compared with those of the SGSB100 and SGSB103 strains because of *budA* and *budB* gene overexpression. The relative quantities of *budA* gene expression (5.96E+02/7.67E+00, 4.40E+02/5.06E+00, 1.80E+03/1.12E+03, and 3.83E+02/3.33E+03) and of *budB* expression (3.30E−01/5.40E-02, 4.15E−01/5.11E-01, 9.42E+02/8.32E+02, and 4.68E+02/4.55E+03) in SGSB100, SGSB103, SGSB104, and SGSB105, respectively, during the lag and stationary phases of cell growth were obtained. In the case of *budA*, all strains exhibited similar values for transcription levels at the lag phase, but the transcription levels at the stationary phase for the SGSB104 and SGSB105 strains were approximately twice the level of the SGSB100 and SGSB103 strains (Fig. 3B). Additionally, relative quantities of *budB* expression in the lag and stationary phases in the SGSB104 and SGSB105 strains reflect transcription levels that were approximately thrice the level of the SGSB100 and SGSB103 strains (Fig. 3C).

Meanwhile, there were no significant differences between any strains with regard to relative quantities of *budC* expression levels at both lag and stationary phases. This result suggests that the expression level of the *budC* gene in the mutants was not affected by overexpression of the *budA* and *budB* genes. The observed changes in gene transcription levels confirm that overexpression of the *budA* and *budB* genes in the SGSB104 and SGSB105 strains resulted in the increase in carbon flux toward 2,3-BDO production (Fig. 3D).

### Confirmation of redistributed 2,3-BDO production in fed-batch fermentation

**Figure 4:**
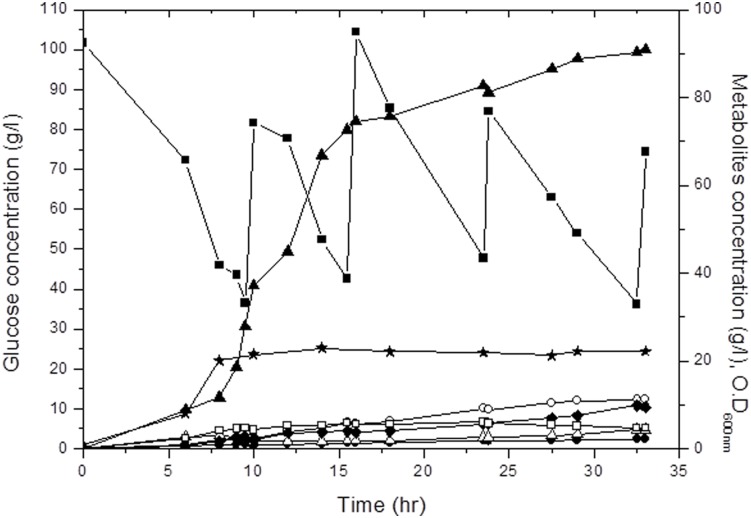
Fed-batch fermentation profile of *K. pneumoniae* strain SGSB105. Closed squares = glucose; closed stars = cell growth; closed triangles = 2,3-BDO; closed diamonds = acetoin; closed circles = lactate; open squares = ethanol; open triangles = acetate; open circles = succinate.

The SGSB105 strain exhibited elevated levels of 2,3-BDO production as well as decreased lactate production in batch fermentation compared with corresponding levels in the SGSB100 strain. RT-PCR results for the *ldhA*, *budA*, *budB*, and *budC* genes confirm that the carbon flux was redistributed toward 2,3-BDO production because of changes at the genetic level. Fed-batch fermentation of the SGSB105 strain was conducted to check the maximal titer of 2,3-BDO produced, yield, and productivity. In the fed-batch fermentation, 1 L of glucose solution (700 g/L) was fed into the bioreactor at a rate of 3.5 ml/min at 30 min intervals. To allow comparison with a previous study [20], culture conditions for pH, agitation speed, and temperature were identical to those in the previous study. During fed-batch fermentation, the 2,3-BDO concentration increased markedly over 14 h (from the log phase to the early stationary phase of cell growth), but the rate of 2,3-BDO production decreased significantly during the late stationary phase. This phenomenon might have occurred because 2,3-BDO is a mixed-growth-associated metabolite [23]. The overall 2,3-BDO production, productivity, and yield of the SGSB105 strain at 33 h were 90 g/L, 2.75 g/L h, and 0.38 g/g, respectively (Fig. 4). Additionally, maximum values of productivity and yield of 2,3-BDO during 6–14 h of fed-batch fermentation were 7.23 g/L h and 0.44 g/g, respectively. In particular, the maximum productivity and yield of the SGSB105 strain was significantly increased by 32% and 16%, respectively, compared with the previous experiment results [20]. In our previous study, by mutant strain which is *budB* and *budA* genes overexpressed, the total and maximum 2,3-BDO productivities (during 6–14 h) were 2.54 and 5.47 g/L h, respectively. *ldhA* deletion led to a decline in lactate production to <1.26 g/L and to formation of byproducts such as acetoin (3.76 g/L), succinic acid (5.61 g/L), ethanol (5.61 g/L), and acetic acid (1.78 g/L) (Fig. 4).

## Discussion

In the present study, carbon flux was redistributed toward 2,3-BDO biosynthesis via metabolic engineering of *K. pneumoniae*. To increase the carbon flux toward 2,3-BDO production, three mutants, namely, a *ldhA* deletion strain (SGSB103), a strain overexpressing *budA* and *budB* (SGSB104), and a strain with *ldhA* deletion and overexpresses *budA* and *budB* (SGSB105), were constructed. The effect of the modifications on 2,3-BDO-biosynthesis was closely observed by batch and fed-batch fermentation and by RT-PCR.

*Klebsiella* species produce neutral metabolites such as 2,3-BDO and ethanol, which prevent acidification of media by acidic metabolites such as lactate, acetate, succinate, and formate. Also, 2,3-BDO synthesis plays a role in regulating the intracellular NAD^+^ balance by consuming NADH [9], [20], [21]. As shown in Fig. 2A, the SGSB100 strain produced lactate as a major acidic product. As biosynthetic pathways for 2,3-BDO and lactic acid consume NADH, these pathways compete with each other. Lactate synthesis from pyruvate consumes one NADH per 1 mole pyruvate, thus remained NADH by deleting *ldhA* gene can be used in 2,3-BDO production [8]. This competition explains the enhanced carbon flux toward 2,3-BDO synthesis in the SGSB103 and SGSB105 strains. During batch fermentation, the carbon flux toward the biomass was similar among strains (SGSB100: 1.88; SGSB103: 1.91; SBSB104: 1.65, and SGSB105: 1.97 g DCW ( = optical densityA600×0.268–0.0489)g/L). Therefore, genetic engineering of *ldhA*, *budA*, and *budB* genes did not inhibit cell growth. Carbon flux toward 2,3-BDO biosynthesis in the SGSB105 strain increased by 33.6% relative to that in the SGSB100 strain (Table 3)**.** Additionally, carbon flux redistribution resulting in lactate production of the total byproducts in the SGSB105 strain decreased to 5% compared with that of the SGSB100 strain (Fig. 2). Moreover, the phenomena in the SGSB105 strain can be explained by the NADH consumption: By blocking the lactate production pathway, the cell needed to consume NADH through other pathways. Thus, the carbon flux toward 2,3-BDO biosynthesis and 2,3-BDO biosynthesis increased because of overexpression of the *budA* and *budB* genes (Fig. 1).

The relative quantities of transcription levels for *ldhA* and for the 2,3-BDO biosynthesis genes (*budA*, *budB*, and *budC*) in the mutant strains were analyzed. Relative quantity data for *budC* in all strains were similar, but those for *budA*, *budB*, and *ldhA* were significantly different. It means that transcription levels of *budC* gene in the mutants were not affected by overexpression of the *budA, budB* and genes. In the 2,3-BDO production, butanediol dehydrogenase encoded by *budC* gene play a role in the reversible reaction of acetoin to 2,3-BDO and regulated the intracellular NAD^+^/NADH balance [22]. Thus, overexpression of *budC* gene does not help to increase 2,3-BDO production, and it was demonstrated by previous study [20]. Meanwhile, transcription levels of the *budA* and *budB* genes in the SGSB104 and SGSB105 strains were essentially retained at the stationary phase of cell growth, in contrast to the levels at the lag phase. 2,3-BDO biosynthesis was elevated because of the increase and retention of the *budA* and *budB* gene transcription levels, indicating that overexpression of the key genes in 2,3-BDO biosynthesis is an important factor in the redistribution of the carbon flux toward 2,3-BDO production.

Many attempts have been made to increase the 2,3-BDO production, productivity, and yield in glucose media in order to develop mutants for industrial use [6], [11], [20], [23]. During the past few years, studies have achieved an approximate 2,3-BDO concentration of 110 g/L 2,3-BDO and a yield of 0.45 g/g [6], [20]. However, the most important characteristic of strains for industrial applications is high productivity in short periods, as it saves time and resources [2]. In our previous study, the total and maximum 2,3-BDO productivities (over 6–14 h) were 2.54 and 5.47 g/L h, respectively [20]. In contrast, the newly constructed SGSB105 strain achieved total and maximum 2,3-BDO productivities (over 6–14 h) of 2.75 and 7.23 g/L h, respectively. These results indicate that the maximum 2,3-BDO productivity of SGSB105 increased by up to 32% compared with that in previous experiments because of redistribution of the carbon flux. Furthermore, through development of the fermentation process can maintain maximum productivity, SGSB105 strain can used as an industrial strain. Additionally, productivity (over 6–14 h) for succinate, lactate, acetoin, and ethanol were 0.47, 0.06, 0.37 and 0.37 g/L h, respectively. And acetate was increased until 9hr and then decreased, it seems that because the activity of the TCA cycle increase [24]. This phenomenon might have consumed residual NADH due to NADH was consumed and NAD^+^ was generated because of accumulation of succinate, acetoin, and ethanol (Fig. 1).

In conclusion, the nonpathogenic SGSB100 strain of *K. pneumoniae* was engineered to redistribute its carbon flux toward 2,3-BDO biosynthesis. Three mutants, namely SGSB103 (with *ldhA* gene deletion), SGSB104 (overexpressing the *budA* and *budB* genes), and SGSB105 (with *ldhA* deletion and overexpressing the *budA* and *budB* genes), were constructed and compared. Among them, the SGSB105 strain exhibited elevated 2,3-BDO production and reduced lactate production. It achieved a maximum 2,3-BDO productivity of 7.23 g/L h in fed-batch fermentation. Through this study, we demonstrated the construction a target microorganism with its carbon flux redistributed toward 2,3-BDO production (SGSB105 strain) through metabolic engineering.
